# Association between serum total testosterone levels and metabolic syndrome among adult women in the United States, NHANES 2011–2016

**DOI:** 10.3389/fendo.2023.1053665

**Published:** 2023-02-09

**Authors:** Chenning Liu, Meiduo Zhao, Yonghua Zhao, Yuanjia Hu

**Affiliations:** ^1^ State Key Laboratory of Quality Research in Chinese Medicine, Institute of Chinese Medical Sciences, University of Macau, Macao, Macao SAR, China; ^2^ Department of Public Health and Medicinal Administration, Faculty of Health Sciences, University of Macau, Macao, Macao SAR, China; ^3^ Department of Epidemiology and Biostatistics, Institute of Basic Medical Sciences Chinese Academy of Medical Sciences, School of Basic Medicine Peking Union Medical College, Beijing, China

**Keywords:** serum total testosterone levels, metabolic syndrome, women, adults, NHANES (National Health and Nutrition Examination Survey)

## Abstract

**Objective:**

To investigate the association between serum total testosterone (TT) levels and metabolic syndrome (MetS) or its components among adult women.

**Methods:**

2,678 women from NHANES 2011-2016 were included in this cross-sectional study. MetS was determined according to the National Cholesterol Education Program Adult Treatment Panel III guidelines. The association between serum TT levels and MetS was evaluated by two logistics regression models and the adjusted restricted cubic spline (RCS). Stratified analysis and sensitive analysis were also conducted.

**Results:**

Continuous TT levels were negatively associated with the occurrence of MetS, and the ORs associated with per SD increase in ln TT were 0.70 (95%CI: 0.58-0.85) in 2011-2014 and 0.56 (95%CI: 0.39-0.79) in 2015-2016 in Model A. High TT group were less likely to have MetS (OR=0.60, 95%CI: 0.45-0.80 in 2011-2014 and OR=0.50, 95%CI: 0.32-0.78 in 2015-2016) when compared to the low TT group. When TT levels were divided into quartiles, TT levels were negatively correlated with the incidence of MetS (*p* for trend < 0.001). Similar trend was observed in Model B. Multivariate-adjusted logistic regression with RCS exhibited that TT had a L-shaped dose–response association with MetS or its components. Interaction analyses revealed that women who were less than 50 years old (OR=0.37, 95%CI: 0.22, 0.63), with depression (OR=0.50, 95%CI: 0.29, 0.87) or being smokers (OR=0.37, 95%CI: 0.23, 0.54) showed lower ORs than those who were over 50 years old (OR=0.66, 95%CI: 0.40, 1.09), without depression (OR=0.59, 95%CI: 0.41, 0.85) or non-smokers (OR=0.59, 95%CI: 0.39, 0.89) when measure the association between ln TT and the occurrence of MetS.

**Conclusions:**

Our study indicated that TT levels are negatively correlated with the occurrence of MetS, with interaction effects of age, smoke behaviors, and depressive status.

## 1 Introduction

Metabolic syndrome (MetS) represents a cluster of metabolic abnormalities. These abnormalities would increase the risk of cardiovascular disease and all-cause mortality (1–3). The prevalence of MetS among American adults has increased substantially, rising from 25.3% in 1988-1994 to 36.9% in 2015-2016 according to the National Health and Nutrition Examination Survey (NHANES) data (4, 5).

Although previous studies have reported that sex hormones are related to an increased cardiometabolic risk and mortality, including MetS, type 2 diabetes mellitus (T2DM), and hypertension (6–8), the roles of these hormones among women are poorly understood. Existing literature has indicated that testosterone deficiency or low serum total testosterone (TT) levels are correlated with an increased risk of MetS or its components in the male population (9–11), and an association between metabolic abnormalities and hyperandrogenism in young women with polycystic ovarian syndrome (PCOS) (12–14). However, the relationships between TT and MetS are inconsistent in the previous studies of women due to different research subjects under certain conditions (such as different ethnic, age group, patient-based samples, post-menopausal women, and small sample sizes) or different research types (7, 15–20).

Thus, this cross-sectional study aims to investigate the association between TT levels and MetS or its components among adult women by using the large and nationally representative survey of the US population from NHANES 2011-2016. This work may provide insightful suggestions on the impact of TT levels on women, which may further be derived to clinical evidence on the controversy of hormonal therapies for women in preventing and managing MetS.

## 2 Methods

### 2.1 Data source

NHANES (21) is a major program carried out by the National Center for Health Statistics (NCHS), which is part of the Centers for Disease Control and Prevention (CDC). It is a series of cross-sectional surveys with every two-year cycle since 1999. This survey used a complex, multistage, stratified probability sampling method to collect nationally representative health statistics on the US population. To produce more reliable and precise statistics, NHANES over-sampled certain population subgroups. Therefore, sample weights were taken into consideration during our data analyses in order to correct for differential selection probabilities, compensate for possible inadequacies in the eligible population, and adjust for non-coverage and non-response.

All NHANES data collection protocols were approved by the National Center for Health Statistics Institutional Review Board and all participants signed an informed consent. NHANES survey data, detailed survey operation manuals, consent documents, and brochures of each period are publicly available on the NHANES website (https://www.cdc.gov/nchs/nhanes).

### 2.2 Study participants

**Figure 1:**
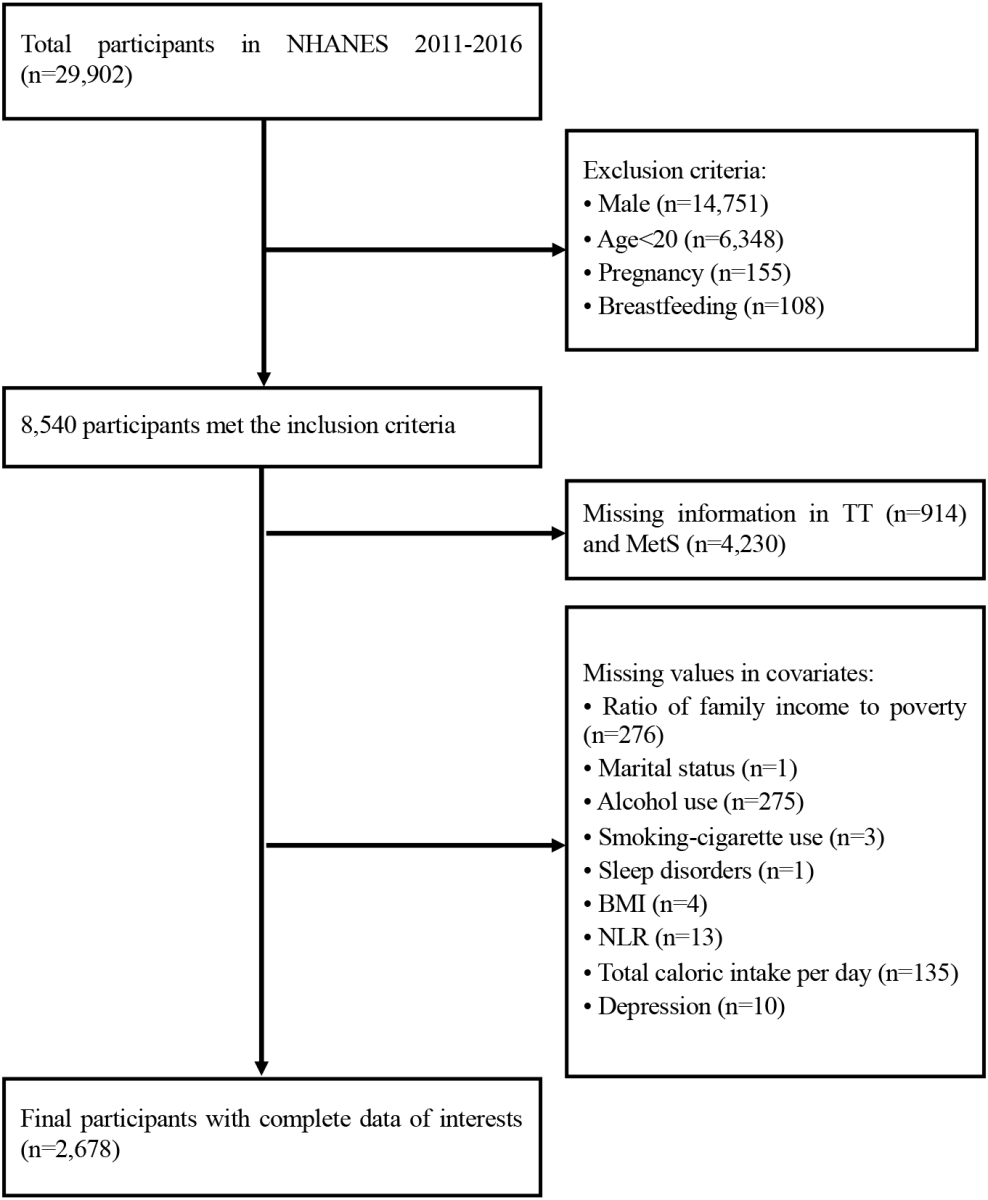
Flowchart of the study design. TT, total serum testosterone level; MetS, metabolic syndrome; BMI, body mass index; NLR, neutrophil-to-lymphocyte ratio.

29,902 participants had completed the interviews and received medical and laboratory testing from the NHANES 2011-2016. We excluded men (n=14,751), women who were less than 20 years (n=6,348), pregnant (n=155) and lactating women (n=108) from our analysis. Those with missing information on TT, MetS or covariates were further excluded. Therefore, the final sample size of our analysis was 2,678 women (**Figure 1**). In order to observe the consistency of research results over different time periods, we divided these datasets into two parts: the data from NHANES 2011-2014 (1,828 women) and the data from NHANES 2015-2016 (850 women).

### 2.3 Diagnosis of MetS

The diagnosis of MetS was determined according to the National Cholesterol Education Program Adult Treatment Panel III guidelines (22). The study participants who met at least 3 of the following criteria were categorized in the MetS group: (1) Elevated waist circumference (>88 cm in women), (2) Elevated serum triglycerides (≥150 mg/dL), (3) Reduced HDL-C (<50 mg/dL in women), (4) High Blood pressure (≥130/85 mm Hg), (5) Elevated serum glucose (≥110 mg/dL).

### 2.4 Serum TT measurement

The serum samples were processed, stored, and transported to the Division of Environmental Health Laboratory Sciences, National Center for Environmental Health for analysis. The concentration of TT was performed by isotope dilution liquid chromatography tandem mass spectrometry (ID-LC-MS/MS) method, which was developed by the CDC. Detailed quality control and quality assurance instructions were discussed in the NHANES Laboratory/Medical Technologists Procedures Manual (LPM).

### 2.5 Covariates assessment

Based on previous studies and clinical plausibility, the following covariates were included in our analysis to reduce potential confounding bias. Information on age, race (Mexican American, other Hispanic, non-Hispanic white, non-Hispanic black, other race-including multi-racial), education (less than high school, high school or equivalent, college or above), marital status (married, widowed, divorced, separated, never married, living with partner), and ratio of family income to poverty (≤1.00, 1.01-3.00, >3.00) were obtained through self-reports of demographic questionnaires. The body mass index (BMI; calculated as weight in kilograms divided by the square of height in meters) was categorized into 4 groups: underweight (<18.5 kg/m2), normal weight (18.5-24.9 kg/m2), overweight (25.0-29.9 kg/m2), obesity (≥30 kg/m2). Alcohol use referred to participants who had at least 12 drinks of any type of alcoholic beverage in any one year. Alcoholic beverages included liquor, beer, wine, wine coolers, and any other type of alcoholic beverage. Information about smokers was that participants who had smoked ≥100 cigarettes in their lifetimes. NHANES has incorporated the Patient Health Questionnaire (PHQ-9) since 2005, which is a self-reported assessment based on nine signs and symptoms of depression over the past two weeks. The score for each item ranged from 0 to 3, and the total score for each participant ranged from 0 to 27. A total score of ≥5 was used as the cut-off for depression. Participants were asked “Ever told doctor had trouble sleeping?”, and those who answered “yes” were considered to have sleep disorders. The total caloric intake per day was estimated based on the types and amounts of foods and beverages (including all types of water) consumed during the 24-hour period prior to the interview (midnight to midnight). Lymphocyte and neutrophil counts were assessed using automated hematology analysis devices and expressed as ×1,000 cells/mm^3^. The neutrophil-to-lymphocyte ratio (NLR) was measured as the ratio of neutrophil count-to-lymphocyte count.

### 2.6 Statistical analysis

Categorical variables were expressed as percentage (%) and compared using the chi-square test or Fisher’s exact test when appropriate. Continuous data with normally distributions were expressed as mean (± standard deviation [SD]) and compared by independent samples t-test. Variables with skewed distributions were expressed as median (interquartile range [IQR]) and compared using the nonparametric Wilcoxon rank sum test. In the data analysis of NHANES 2011-2014, we used two logistics regression models to investigate the association between serum TT levels and MetS among women. Model A only adjusted for age, race, and BMI. Model B further adjusted for other demographic characteristics, lifestyle variables, and some related health indicators: education level, marital status, ratio of family income to poverty, alcohol use, smoking-cigarette use, depression, sleep disorders, total caloric intake per day, and NLR. We also evaluated the presence of nonlinear dose-response relationships between ln-transformed TT (ln TT) and MetS or its components by the adjusted restricted cubic spline (RCS) with three knots (percentile 25, 50, and 75). We measured the interaction of TT with various clinical parameters with product interaction terms. To investigate the modified effect of age, depression, smoking status and TT on the incidence of MetS, we stratified the study population by age (less than 50 years or above), depression status (No or Yes) and smoke (No or Yes). We also performed sensitive analysis after excluding participants who were taking antidiabetic drugs, hypertension medication or lipid-lowering medication or women with ovary removed, and comparing results across samples over different time periods. For the female population from NHANES 2015-2016, we also used two logistics regression models with increasing degrees of adjustment to evaluate the association between TT levels and the odds ratio of MetS. Meanwhile, we also stratified women by age (less than 50 years or above) to determine the relationship between TT levels and MetS at different age stages. All reported *p* values were 2-sided, and the significance level was set at 0.05. R software (Version 4.1.2) was used for statistical analysis.

## 3 Results

### 3.1 Characteristics of the participants

The baseline characteristics of the study population with weighted estimates were presented in **Table 1**. Women were ranged from 20 to 80 years, with an average age of 49.52 ± 16.86 years. The weighted distribution of races was as follows: 6.9% of the participants were Mexican American, 5.4% were other Hispanic, 70.0% were non-Hispanic White, 10.5% were non-Hispanic Black, and 7.1% were others. The distribution of age, education level, marital status, ratio of family income to poverty, BMI, alcohol use, smoking-cigarette use, depression, sleep disorders, NLR, and TT levels were significantly different between the MetS group and the non-MetS group (all *p*<0.05). The median (IQR) of TT levels in MetS group were 17.80 [12.20, 24.01] ng/dL, which were lower than that in non-MetS group (22.20 [15.50, 30.30] ng/dL). In general, TT levels were negative correlated with age (**Figure S1**).

**Table 1:** Characteristics of study participants in the NHANES 2011-2016^*^.

Variables	Overall (n=2,678)	MetS (n=813)	Non-MetS (n=1,865)	*p*-value
Weighted sample size	78,487,974	22,829,762	55,658,212	
Age, years	49.52 ± 16.86	55.60 ± 14.52	47.02 ± 17.12	<0.001
Race, n (%)				0.150
Mexican American	344 (6.9)	123 (7.9)	221 (6.5)	
Other Hispanic	303 (5.4)	101 (4.9)	202 (5.7)	
Non-Hispanic White	1125 (70.0)	358 (71.9)	767 (69.2)	
Non-Hispanic Black	572 (10.5)	167 (9.3)	405 (11.0)	
Other Race	334 (7.1)	64 (5.9)	270 (7.6)	
Education level, n (%)				0.003
Less than high school	522 (12.4)	220 (17.1)	302 (10.5)	
High school or equivalent	533 (20.1)	178 (22.1)	355 (19.3)	
College or above	1623 (67.5)	415 (60.8)	1208 (70.2)	
Marital status, n (%)				<0.001
Married	1225 (53.3)	381 (53.4)	844 (53.3)	
Widowed	282 (8.3)	124 (13.3)	158 (6.3)	
Divorced	334 (11.7)	118 (13.7)	216 (10.9)	
Separated	106 (2.2)	39 (2.2)	67 (2.2)	
Never married	530 (16.5)	100 (9.8)	430 (19.2)	
Living with partner	201 (8.0)	51 (7.6)	150 (8.2)	
Ratio of family income to poverty,n (%)				0.003
≤1.00	662 (15.9)	227 (17.4)	435 (15.3)	
1.01-3.00	1065 (35.5)	362 (41.1)	703 (33.2)	
>3.00	951 (48.6)	224 (41.4)	727 (51.5)	
BMI (kg/m^2^), n (%)				<0.001
<18.5	54 (2.1)	1 (0.1)	53 (2.9)	
18.5-24.9	731 (27.8)	49 (5.7)	682 (36.9)	
25.0-29.9	743 (28.6)	201 (24.6)	542 (30.2)	
≥30.0	1150 (41.5)	562 (69.6)	588 (30.0)	
Alcohol use, n (%)	1683 (70.6)	451 (64.2)	1232 (73.2)	<0.001
Smoking-cigarette use, n (%)	950 (39.7)	349 (48.5)	601 (36.1)	0.001
Depression, n (%)	777 (27.1)	322 (37.9)	455 (22.7)	<0.001
Sleep disorders, n (%)	839 (33.5)	328 (44.0)	511 (29.2)	<0.001
Total caloric intake per day (kcal, day), n (%)				0.729
<1550	1086 (37.0)	349 (37.4)	737 (36.8)	
1550-1972	623(25.5)	170 (23.3)	453 (26.4)	
1973-2554	580 (22.9)	184 (23.9)	396 (22.5)	
≥2555	389 (14.7)	110 (15.4)	279 (14.4)	
NLR	2.14 ± 1.11	2.34 ± 1.35	2.05 ± 0.99	<0.001
Total testosterone (ng/dL)	20.50 [14.50, 28.90]	17.80 [12.20, 24.01]	22.20 [15.50, 30.30]	<0.001

^*^All estimates accounted for sample weights and complex survey designs, and means and percentages were adjusted for survey weights of NHANES. MetS, metabolic syndrome; BMI, body mass index; NLR, neutrophil-to-lymphocyte ratio.

### 3.2 Correlation between TT and the incidence of MetS or its components

**Figure 2:**
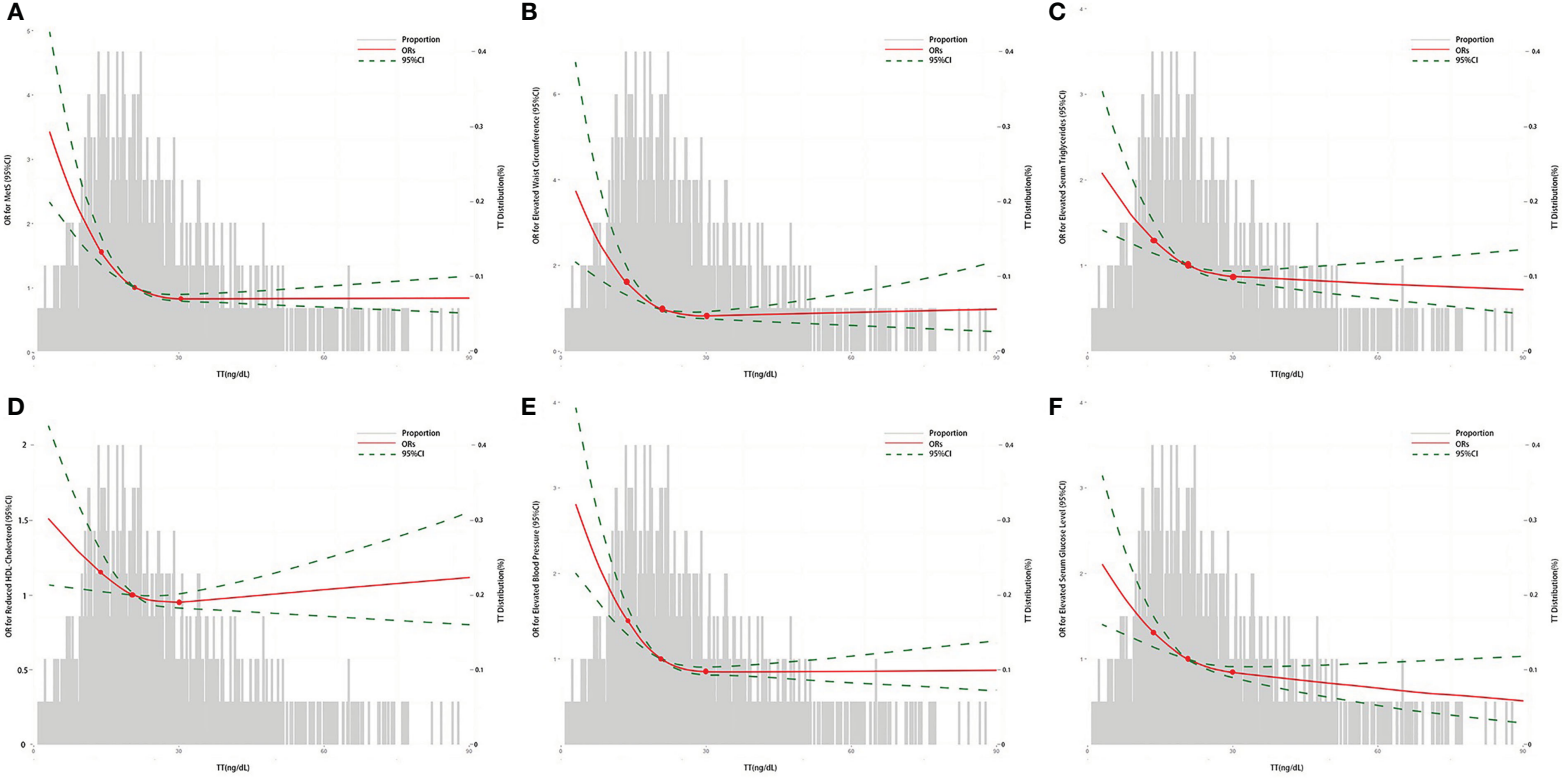
The association between TT and the ORs for MetS or its components in women by logistic regression. **(A)** The association between TT and the ORs for MetS; **(B)** The association between TT and the ORs for elevated waist circumference; **(C)** The association between TT and the ORs for elevated serum triglycerides; **(D)** The association between TT and the ORs for reduced HDL-Cholesterol; **(E)** The association between TT and the ORs for elevated blood pressure; **(F)** The association between TT and the ORs for elevated serum glucose level. The curves represent the adjusted ORs of MetS or its components by ln-transformed TT levels. The dose response association was estimated by using nonlinear dose-response relationships between ln TT and MetS or its components by the adjusted restricted cubic spline with three knots (percentile 25, 50, and 75). Model adjusted for age, race, education level, marital status, ratio of family income to poverty, BMI, alcohol use, smoking-cigarette use, depression, sleep disorders, total caloric intake per day, and NLR. TT, total serum testosterone level; MetS, metabolic syndrome; BMI, body mass index; NLR, neutrophil-to-lymphocyte ratio; OR, odds ratio.

**Table 2** showed the results from the multivariate regression models between TT levels and MetS in women. In Model A, continuous TT levels were negatively associated with the occurrence of MetS, and the ORs associated with per SD increase in ln TT were 0.70 (95%CI: 0.58-0.85) in 2011-2014 and 0.56 (95%CI: 0.39-0.79) in 2015-2016 after adjusting for age, race, and BMI. Similarly, women in high TT group were less likely to have MetS (OR=0.60, 95%CI: 0.45-0.80 in 2011-2014 and OR=0.50, 95%CI: 0.32-0.78 in 2015-2016) when compared to the low TT group. When TT levels were divided into quartiles, the results in 2011-2014 and 2015-2016 both suggested that TT levels were negatively correlated with the incidence of MetS (*p* for trend < 0.001). Women in Quartile 4 (30.10-575.00 ng/dL) in 2011-2014, Quartile 3 (20.40-28.10 ng/dL) and Quartile 4 (28.30-444.00 ng/dL) in 2015-2016 showed statistically significant lower odds of MetS. Similar trend that a statistically lower occurrence of MetS was observed among women with high TT levels were obtained in Model B: OR=0.70 (95%CI: 0.56-0.87) in 2011-2014 and OR=0.57 (95%CI: 0.33-0.99) in 2015-2016 per SD increase in ln TT; OR=0.61 (95%CI: 0.45-0.81) in 2011-2014 and OR=0.47 (95%CI: 0.24, 0.92) in 2015-2016 in high TT group. Multivariate-adjusted logistic regression with RCS exhibited that TT levels had a L-shaped dose–response association with MetS or its components, when analyzed the association between TT levels and the incidence of MetS or its components (**Figure 2**).

**Table 2:** The association between TT and MetS in women^a^.

TT	Model A		Model B	
	2011-2014	2015-2016	2011-2014	2015-2016
**As continuous (ln, per SD)**	0.70 (0.58, 0.85)^***^	0.56 (0.39, 0.79)^**^	0.70 (0.56, 0.87)^**^	0.57 (0.33, 0.99)^*^
By cut-off				
Low	Ref.		Ref.	
High	0.60 (0.45, 0.80)^***^	0.50 (0.32,0.78)^**^	0.61 (0.45, 0.81)^**^	0.47 (0.24, 0.92)^*^
Interquartile (ng/dL) ^b^				
Quartile 1	Ref.		Ref.	
Quartile 2	0.88 (0.56, 1.37)	0.62 (0.37, 1.06)	0.88 (0.54, 1.44)	0.68 (0.23, 1.99)
Quartile 3	0.68 (0.41, 1.11)	0.41 (0.20, 0.85)^*^	0.66 (0.38, 1.15)	0.48 (0.11, 2.10)
Quartile 4	0.55 (0.36, 0.82)^**^	0.36 (0.22, 0.61)^**^	0.55 (0.35, 0.88)^**^	0.39 (0.12, 1.23)

^a^The association between TT and MetS in women was presented by the ORs (95%CI). ^b^Interquartile (ng/dL) for 2011-2014: Quartile 1, 1.02-13.80; Quartile 2, 13.84-20.70; Quartile 3, 20.71-30.06, Quartile 4, 30.10-575.00; Interquartile (ng/dL) for 2015-2016: Quartile 1, 1.52-14.10; Quartile 2, 14.20-20.30; Quartile 3, 20.40-28.10; Quartile 4, 28.30-444.00; Model A: Adjusted for age, race, and BMI. Model B: Adjusted for age, race, education level, marital status, ratio of family income to poverty, BMI, alcohol use, smoking-cigarette use, depression, sleep disorders, total caloric intake per day, and NLR. TT, total serum testosterone level; MetS, metabolic syndrome; BMI, body mass index; NLR, neutrophil-to-lymphocyte ratio; OR, odds ratio; CI, confidence interval. ^*^p ≤ 0.05; ^**^p ≤ 0.01; ^***^p ≤ 0.001.

### 3.3 Stratified analyses by potential effect modifiers

**Figure 3:**
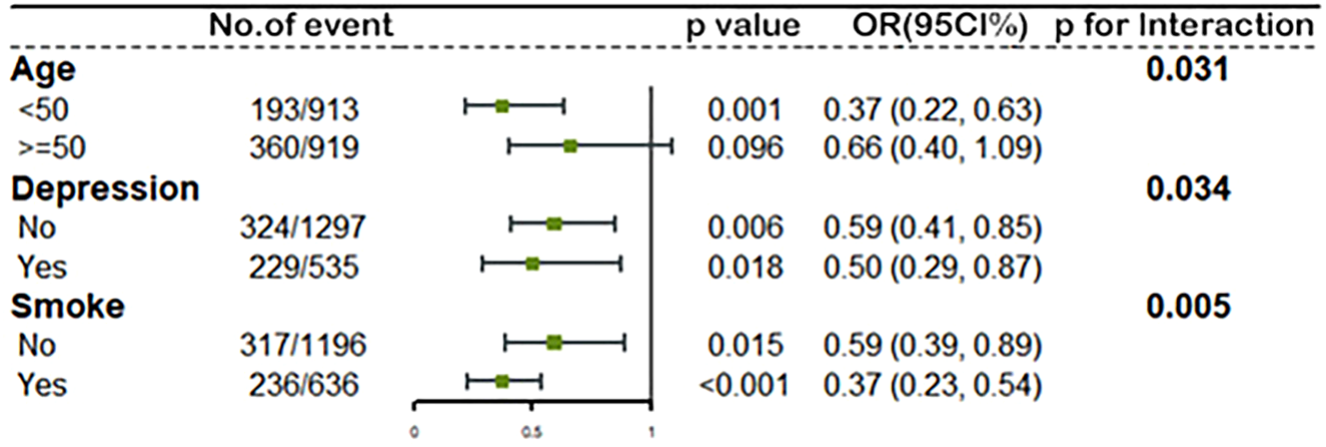
The association between ln TT and MetS stratified by age, depression status and smoke by logistic regression. All models adjusted for race, education level, marital status, ratio of family income to poverty, BMI, alcohol use, sleep disorders, total caloric intake per day, and NLR. The green spot and lines represent the odds ratios (OR) and 95% confidence interval of OR. Ln TT, ln-transformed total serum testosterone level; MetS, metabolic syndrome; BMI, body mass index; NLR, neutrophil-to-lymphocyte ratio.

To investigate the potential modified effect of TT levels and the other factors on the occurrence of MetS, we stratified the study population with the mentioned modifiers. Interaction analyses revealed that TT levels were significantly associated with MetS for the following factors: age, depression, and smoking behaviors (all *p* for interaction < 0.05). As shown in **Figure 3**, women who were less than 50 years old (OR=0.37, 95%CI: 0.22, 0.63), with depression (OR=0.50, 95%CI: 0.29, 0.87) or being smokers (OR=0.37, 95%CI: 0.23, 0.54) showed lower ORs than those who were over 50 years old (OR=0.66, 95%CI: 0.40, 1.09), without depression (OR=0.59, 95%CI: 0.41, 0.85) or non-smokers (OR=0.59, 95%CI: 0.39, 0.89) when we measured the association between ln TT and the occurrence of MetS. **Table 3** showed different trends between the concentration of TT and the occurrence of MetS from the multivariate regression analysis when women were divided into two groups according to the average menopausal age (50 years). It suggested that TT levels were negatively associated with the occurrence of MetS among women who were less than 50 years during different time periods. Continuous TT levels were negatively associated with the occurrence of MetS, and the ORs associated with per SD increase in ln TT were both 0.54 (95%CI: 0.32-0.92) in 2011-2014 and 2015-2016. Meanwhile, women in high TT group were less likely to have MetS (OR=0.37, 95%CI: 0.22-0.63 in 2011-2014 and OR=0.28, 95%CI: 0.12-0.67 in 2015-2016). However, for women ≥50 years, there was no statistically significant difference between high TT group and low TT group (*p* > 0.05). In addition, when the TT levels were divided into quartiles, there was no statistical difference in the occurrence of MetS between the groups, except for women in Quartile 4 who had a decreased risk of MetS than that in Quartile 1.

**Table 3:** The association between TT and MetS in female population from different age stages (<50 years and ≥50 years) ^a^.

TT	<50 years		≥50 years	
	2011-2014	2015-2016	2011-2014	2015-2016
**As continuous (ln, per SD)**	0.54 (0.32, 0.92)^*^	0.54 (0.32, 0.92)^*^	0.74 (0.58, 0.96)^*^	0.71 (0.43, 1.16)
By cut-off				
Low	Ref.		Ref.	
High	0.37 (0.22, 0.63)^***^	0.28 (0.12, 0.67)^*^	0.66 (0.40, 1.09)	0.70 (0.30, 1.65)
Interquartile				
Quartile 1	Ref.		Ref.	
Quartile 2	0.84 (0.43, 1.63)	0.77 (0.12, 5.05)	0.93 (0.60, 1.44)	0.35 (0.07, 1.84)
Quartile 3	0.26 (0.12, 0.54)^***^	0.23 (0.22, 2.35)	0.75 (0.35, 1.58)	0.64 (0.13, 3.24)
Quartile 4	0.48 (0.23, 1.00)^*^	0.29 (0.03, 2.62)	0.53 (0.31, 0.93)^*^	0.42 (0.10, 1.71)

a The association between TT and MetS in women was presented by the ORs (95%CI). All models adjusted for race, education level, marital status, ratio of family income to poverty, BMI, alcohol use, smoking-cigarette use, depression, sleep disorders, total caloric intake per day, and NLR. TT, total serum testosterone level; MetS, metabolic syndrome; BMI, body mass index; NLR, neutrophil-to-lymphocyte ratio; OR, odds ratio; CI, confidence interval. ^*^p ≤ 0.05, ^***^p ≤ 0.001.

### 3.4 Sensitive analysis

Given the observation that the concentration of TT was prognostic for women with MetS, a sensitivity analysis was investigated. We conducted sensitivity analyses after excluding the participants who were taking antidiabetic drugs, hypertension medication or lipid-lowering medication or those women with ovary removed, and comparing results across samples over different time periods, and similar results were obtained (**Tables 2**, **3**, **Figure S2**, and **Figure S3**).

## 4 Discussion

We used the datasets from NHANES 2011-2016 for multi-dimensional analysis in order to obtain reliable results of the relationship between TT and Mets in the adult female population. In this large cross-sectional study of nationally representative US adult women, we found that TT levels negatively correlated with the occurrence of MetS, which was in accordance with previous observational studies in the male population (8, 9, 23, 24). In analyzing the data of NHANES 2011-2014, we used 2 models with increasing degrees of adjustment for confounding factors, and a similar trend that a statistically lower occurrence of MetS was observed among women with high TT levels was obtained. Meanwhile, these trends were in agreement with the sensitive analysis results of the participants after excluding those who were taking antidiabetic drugs, hypertension medication or lipid-lowering medication or those women with ovary removed. And similar results were obtained from the datasets of NHANES 2015-2016. However, there was no statistically significant difference between high TT group and low TT group among women ≥50 years.

In our study, median testosterone in women was 20.50 [14.50, 28.90] ng/dL, and TT levels were negative correlated with age. Our study suggested that TT had a L-shaped dose–response association with MetS and its components, and the relationships between TT levels and the outcome were not exactly the same when the concentration of TT was in different quartile range. Although these results are consistent with some previous studies, the discrepancies in different studies may be associated with different genders and ages. TT levels vary greatly in different genders, ages, or conditions. The normal physiologic range of TT in male population is 450-600 ng/dL (25, 26), and the cut-off value for the diagnosis of testosterone deficiency is 300 ng/dL, which may need testosterone replacement therapy (26). In addition, a reduction of serum TT levels is associated with aging in both men and women (27–29).

Androgen excess is one of the main characteristics of PCOS and is present in 70% of diagnosed women (30, 31). PCOS, which affects 5-20% of women of reproductive age worldwide, is associated with an increased risk of metabolic abnormalities, especially among those women who also show hyperandrogenism (32–34). Therefore, women with PCOS may interfere with the research results. These biases are found in previous studies focusing on the associations between TT and MetS. Cross-sectional relationships between endogenous androgens, sex hormone-binding globulin (SHBG), and MetS were summarized in a meta-analysis, which aimed to compare the relationships in terms of sex differences (15). This study comprised 13,974 men and 4,063 women on the relationship between TT and MetS, and the results showed a reduced MetS risk with higher TT levels in male population. In contrast, women who were in the highest tertile of TT had an increased risk of incident MetS compared with women in the lowest tertile (RR 1.68, 95% CI 1.15, 2.45). However, of the 15 studies which were included in this meta-analysis, 13 studies did not adjust for age and 9 studies focused on elderly women (55 years old and above). In addition, 6 studies are focused on women with PCOS, who have an increased risk of metabolic syndrome. All these biases may affect the results.

In terms of the MetS components, the relationships showed a L-shaped dose–response between TT levels and all components in our study, which was consistent with most previous results in male population (11, 35). An analysis from NHANES 2011-2012 reported the inverse relationship between TT levels and MetS components, and indicated that only serum triglycerides, HDL-C, and serum glucose were correlated with TT levels (17). The differential results between TT levels and MetS components may be due to the difference of the sample size.

Given that previous studies on the relationship between TT and MetS in certain conditions or different age groups had obtained different results, we expanded our study by stratified analyses to demonstrate that TT had different impacts on MetS at different ages. Globally, the mean age of natural menopause is around 50 years, with remarkably little geographic variation (36, 37). Therefore, we stratified women according to the average menopausal age, and different trends were observed. This research showed that there was no statistically significant difference between the two groups divided by serum TT levels among women aged 50 or above, which was consistent with that of some studies on postmenopausal women (38, 39). A multicenter prospective cohort study from the Netherlands Study of Depression in Older Persons suggested that there was no association between TT and MetS or its components in women aged between 60 and 93 years, regardless of whether major depressive disorder was adjusted for (40). These results may mean that the increased prevalence of the MetS after menopause may be related to estrogen deficiency caused by ovarian failure or the change of other androgens, rather than the effect of TT.

However, the research has several limitations too. In this research, we did not include evaluation of albumin and sex hormone binding globulin levels to calculate free androgen index or bioavailable TT, and did not consider other androgens, such as dehydroepiandrosterone and androstenedione. Besides, the cross-sectional nature of the survey could not determine causal inference. In addition, although we controlled for wide ranges of major potential confounders including demographics, self-reported diseases, medications, lifestyles, and dietary risk factors in the multivariable logistic models, there might be residual confounding from unmeasured factors which could have impacted the effect size estimates. Unfortunately, women with PCOS cannot be excluded from the datasets in this study, which may cause bias.

To our knowledge, this is the first large scale epidemiological study that used nationally representative US population data and examined the association between TT levels and the incidence of Mets or its components among adult women of all ages by multi-dimensional analysis which includes stratified analysis and sensitive analysis. In addition, our results are robust after adjustment for a wide spectrum of potential confounders.

## 5 Conclusions

The present cross-sectional study indicated that TT levels were negatively correlated with the occurrence of MetS or its components, and TT had a L-shaped dose-response association with MetS or its components. In addition, the trend of negative correlation between TT levels and the occurrence of MetS was more obvious among women who were less than 50 years old, with depression or being smokers than those who were over 50 years old, without depression or non-smokers. However, different trends were observed when we stratified women according to the average menopausal age. There was no statistically significant association between serum TT levels and the occurrence of MetS among women aged 50 or above. Future studies are necessary to determine the cut-off value for abnormal TT levels in women and the impacts of TT on Mets in different ranges.

## Data availability statement

The original contributions presented in the study are included in the article/**Supplementary Material**. Further inquiries can be directed to the corresponding author.

## Ethics statement

The studies involving human participants were reviewed and approved by National Center for Health Statistics Institutional Review Board. The patients/participants provided their written informed consent to participate in this study.

## Author contributions

YH contributed to the conception and design of this study. CL and MZ organized the database and CL wrote the first draft of the manuscript. MZ performed the statistical analysis. YZ put forward academic suggestions and revised the draft. All authors contributed to the article and approved the submitted version.

## Supplementary material

Supplementary Figure 1 Distribution of TT by age. The spot represents each observation, and the red line is estimated by spearman correlation.TT, total serum testosterone level.

Supplementary Figure 2 The association between TT and MetS among participants who were not taking antidiabetic drugs, hypertension medication or lipid-lowering medication with logistic regression. The green spot and lines represent the odds ratios (OR) and 95% confidence interval of OR. Model adjusted for race, education level, marital status, ratio of family income to poverty, BMI, alcohol use, smoking-cigarette use, depression, sleep disorders, total caloric intake per day, and NLR. TT, total serum testosterone level; MetS, metabolic syndrome; BMI, body mass index; NLR, neutrophil-to-lymphocyte ratio.

Supplementary Figure 3 The association between TT and MetS among participants without ovary removed by logistic regression. The green spot and lines represent the odds ratios (OR) and 95% confidence interval of OR. Model adjusted for race, education level, marital status, ratio of family income to poverty, BMI, alcohol use, smoking-cigarette use, depression, sleep disorders, total caloric intake per day, and NLR. TT, total serum testosterone level; MetS, metabolic syndrome; BMI, body mass index; NLR, neutrophil-to-lymphocyte ratio.
