# Supplementary figures and images for: Association between serum total testosterone levels and metabolic syndrome among adult women in the United States, NHANES 2011–2016
Source: Front Endocrinol (Lausanne). 2023 Feb 9;14:1053665. doi: 10.3389/fendo.2023.1053665 (PMC9946982; doi:10.3389/fendo.2023.1053665)

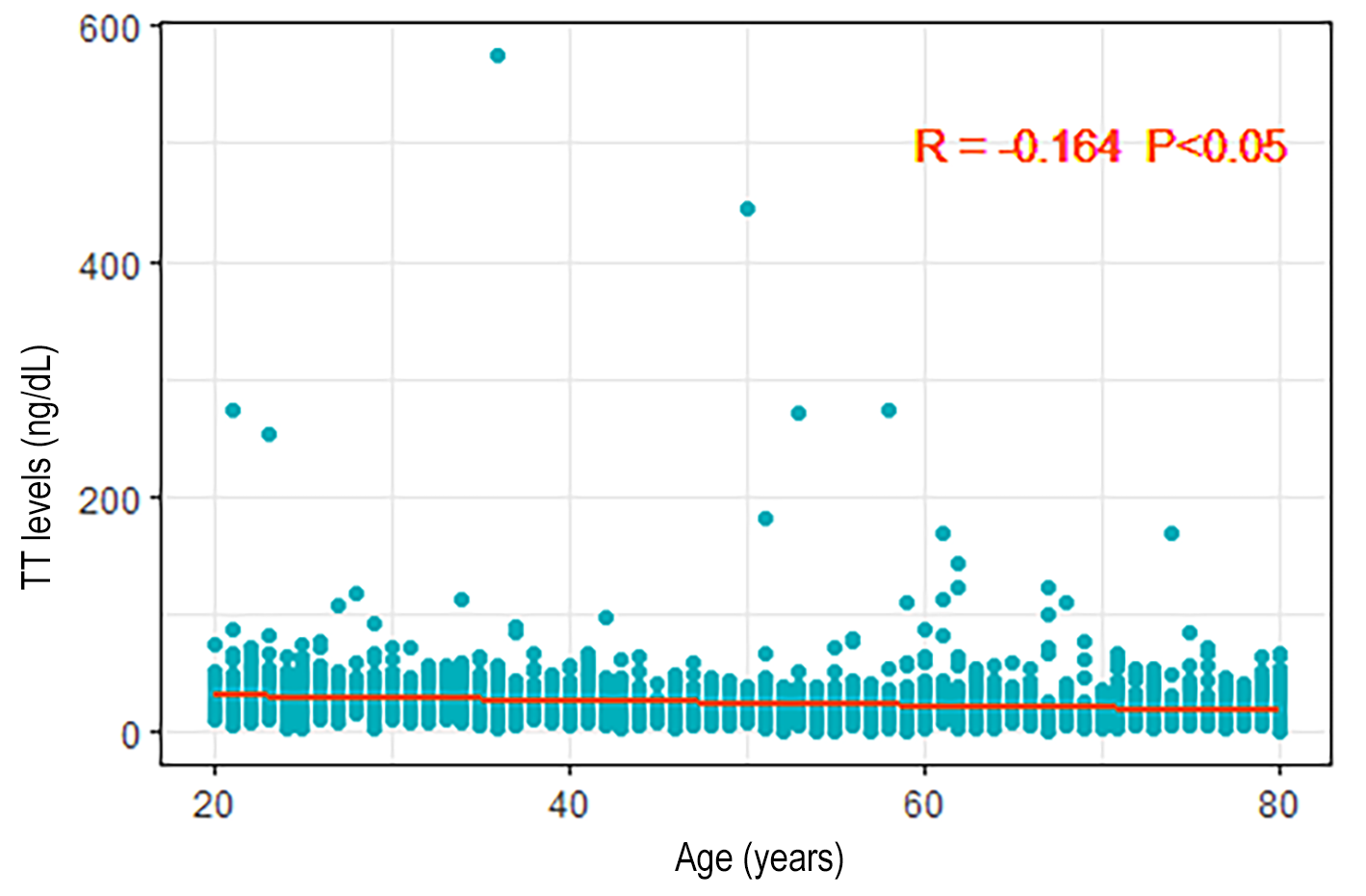

Supplement: Supplementary Figure 1 — Distribution of TT by age. The spot represents each observation, and the red line is estimated by spearman correlation.TT, total serum testosterone level. [file DataSheet_1.zip › Fig.S1.tif]

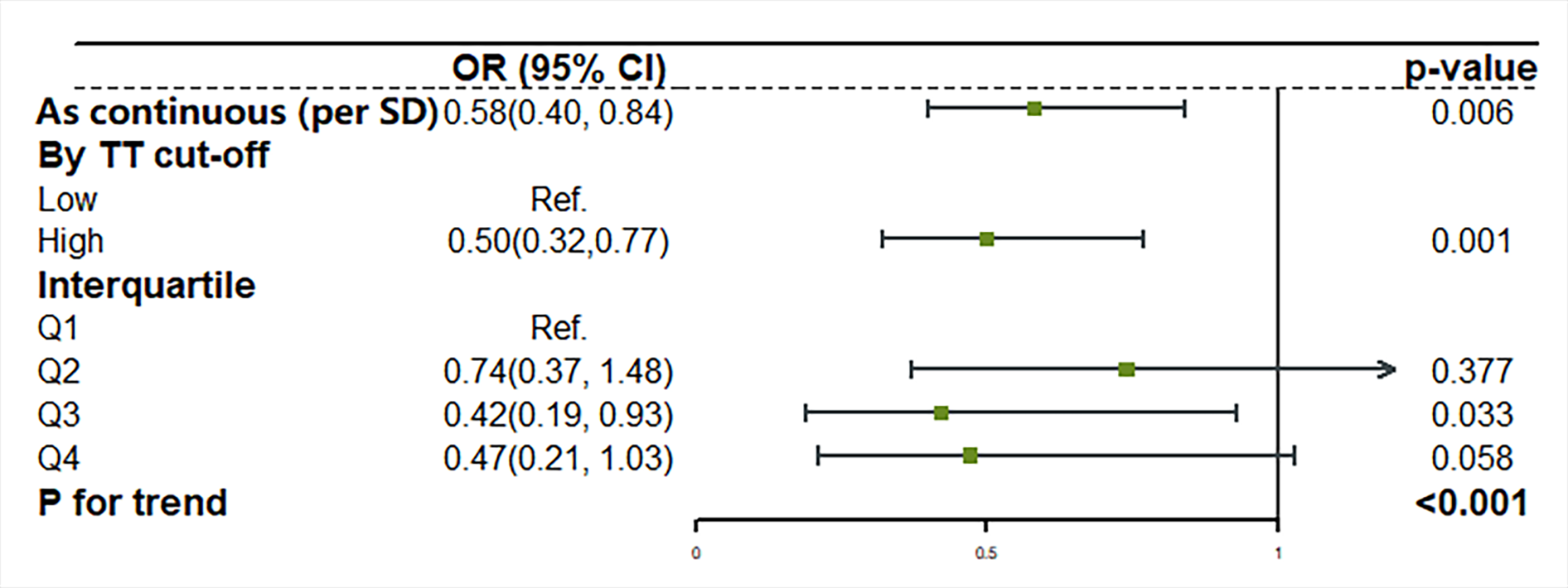

Supplement: Supplementary Figure 1 — Distribution of TT by age. The spot represents each observation, and the red line is estimated by spearman correlation.TT, total serum testosterone level. [file DataSheet_1.zip › Fig.S2.tif]

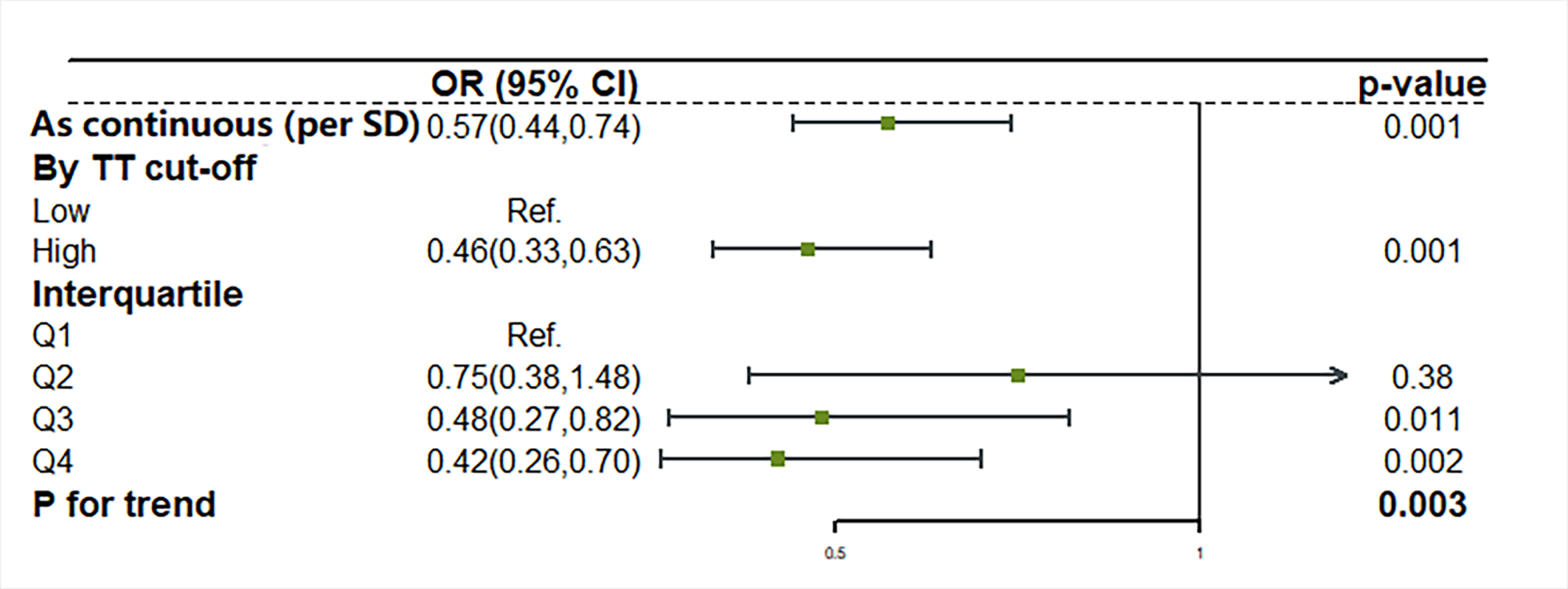

Supplement: Supplementary Figure 1 — Distribution of TT by age. The spot represents each observation, and the red line is estimated by spearman correlation.TT, total serum testosterone level. [file DataSheet_1.zip › Fig.S3.tif]
